# Pandrug-resistant *Acinetobacter baumannii* from different clones and regions in Mexico have a similar plasmid carrying the *bla*_OXA-72_ gene

**DOI:** 10.3389/fcimb.2023.1278819

**Published:** 2023-12-18

**Authors:** José Luis Fernández-Vázquez, Ismael Luis Hernández-González, Santiago Castillo-Ramírez, Ma Dolores Jarillo-Quijada, Catalina Gayosso-Vázquez, Valeria Eréndira Mateo-Estrada, Rayo Morfín-Otero, Eduardo Rodríguez-Noriega, José Ignacio Santos-Preciado, María Dolores Alcántar-Curiel

**Affiliations:** ^1^ Laboratorio de Infectología, Microbiología e Inmunología Clínica, Unidad de Investigación en Medicina Experimental, Facultad de Medicina, Universidad Nacional Autónoma de México, Ciudad de México, Mexico; ^2^ Programa de Genómica Evolutiva, Centro de Ciencias Genómicas, Universidad Nacional Autónoma de México, Cuernavaca, Mexico; ^3^ Hospital Civil de Guadalajara “Fray Antonio Alcalde” e Instituto de Patología Infecciosa y Experimental, Centro Universitario de Ciencias de la Salud, Universidad de Guadalajara, Guadalajara, Mexico

**Keywords:** *Acinetobacter baumannii*, pandrug-resistant, *bla*_OXA-72_ gene, plasmid, Mexico

## Abstract

**Background:**

Multidrug-resistant *Acinetobacter baumannii* is a common hospital-acquired pathogen. The increase in antibiotic resistance is commonly due to the acquisition of mobile genetic elements carrying antibiotic resistance genes. To comprehend this, we analyzed the resistome and virulome of Mexican *A. baumannii* multidrug-resistant isolates.

**Methods:**

Six clinical strains of *A. baumannii* from three Mexican hospitals were sequenced using the Illumina platform, the genomes were assembled with SPAdes and annotated with Prokka. Plasmid SPAdes and MobRecon were used to identify the potential plasmid sequences. Sequence Type (ST) assignation under the MLST Oxford scheme was performed using the PubMLST database. Homologous gene search for known virulent factors was performed using the virulence factor database VFDB and an *in silico* prediction of the resistome was conducted via the ResFinder databases.

**Results:**

The six strains studied belong to different STs and clonal complexes (CC): two strains were ST208 and one was ST369; these two STs belong to the same lineage CC92, which is part of the international clone (IC) 2. Another two strains were ST758 and one was ST1054, both STs belonging to the same lineage CC636, which is within IC5. The resistome analysis of the six strains identified between 7 to 14 antibiotic resistance genes to different families of drugs, including beta-lactams, aminoglycosides, fluoroquinolones and carbapenems. We detected between 1 to 4 plasmids per strain with sizes from 1,800 bp to 111,044 bp. Two strains from hospitals in Mexico City and Guadalajara had a plasmid each of 10,012 bp pAba78r and pAba79f, respectively, which contained the *bla*_OXA-72_ gene. The structure of this plasmid showed the same 13 genes in both strains, but 4 of them were inverted in one of the strains. Finally, the six strains contain 49 identical virulence genes related to immune response evasion, quorum-sensing, and secretion systems, among others.

**Conclusion:**

Resistance to carbapenems due to pAba78r and pAba79f plasmids in Aba pandrug-resistant strains from different geographic areas of Mexico and different clones was detected. Our results provide further evidence that plasmids are highly relevant for the horizontal transfer of antibiotic resistance genes between different clones of *A. baumannii.*

## Introduction

*Acinetobacter baumannii* is a Gram-negative, non-spore-forming, strictly aerobic, non-flagellated exhibiting twitching motility catalase positive, oxidase negative bacterium (Wilharm et al., 2013). It is an opportunistic pathogen that can colonize the skin and cause various healthcare-associated infections (HAIs); predominantly pneumonia and catheter-associated bacteremia but can also cause soft tissue and urinary tract infections (Wong et al., 2017). Infections caused by *A. baumannii* commonly occur in immunocompromised patients with a high incidence in hospital settings. These infections contribute to attributable mortality rates that range from 5% in general hospital wards to as high as 54% in ICUs (Shahryari et al., 2021).

Due to its intrinsic resistance to numerous antimicrobial agents and its ability to efficiently acquire various resistance mechanisms, *A. baumannii* isolates are frequently multi-drug-resistant (MDR) or extensively drug-resistant (XDR). In this regard, carbapenem-resistant *A. baumannii* (CRAB) has been recognized as a critical priority pathogen in the World Health Organization´s priority list of antibiotic-resistant bacteria (Kabic et al., 2023). This species belongs to the so-called “ESKAPE group”, which also includes *Enterococcus faecium*, *Staphylococcus aureus*, *Klebsiella pneumoniae*, *Pseudomonas aeruginosa*, and *Enterobacter* species (Karlowsky et al., 2017). These bacteria not only cause the majority of HAIs but also represent different paradigms of pathogenicity, transmission, and antimicrobial resistance, posing a serious threat to hospitals (Jie et al., 2021).

One significant mechanism of carbapenem resistance involves the hydrolysis of carbapenems by carbapenemase enzymes, which are predominantly encoded on plasmids and, in some cases, on the chromosome, such as oxacillinases. The enzymes carbapenemases exhibited a high degree of transmissibility (Nordmann and Poirel, 2013; Nordmann and Poirel, 2019; Graña-Miraglia et al., 2020). Among carbapenem-resistant *A. baumannii* strains from Latin American countries, the most widely disseminated carbapenemases belong to the class D enzymes (Rodríguez et al., 2018), which include OXA-23, OXA-58, OXA-72, OXA-143 and OXA-253; however, NDM-1, VIM-1, IMP-1, and IMP-10 have also been detected (Nordmann and Poirel, 2019).

Several studies have demonstrated that *A. baumannii* has a natural ability to incorporate exogenous DNA through horizontal gene transfer (HGT), including antibiotic resistance determinants (Traglia et al., 2014). The frequent identification of foreign DNA in its genome explains the genomic plasticity of this pathogen (Lee et al., 2017); HGT is the main mechanism for acquiring new traits, such as antimicrobial resistance genes, allowing the survival of bacterial species with high genetic plasticity (Partridge et al., 2018). Currently, there is a particular interest in studying plasmids carrying genes encoding OXA-type beta-lactamases, which are the primary mechanism of carbapenem resistance in *A. baumannii* (Salgado-Camargo et al., 2020).

Whole-genome sequencing techniques, combined with bioinformatic tools, have been of great help not only to identify the entire gene background that a strain possesses but also to determine different replicons and the genes encoded within them (Graña-Miraglia et al., 2020). Here, we sequenced six Mexican strains of *A. baumannii* sampled from three hospitals to obtain information about the resistome, virulome, and structural dynamics of antimicrobial resistance plasmids.

## Materials and methods

### Study design, settings, and isolates

We selected six *A. baumannii* strains isolated from patients with healthcare-associated infections (HAIs) from three tertiary referral hospitals in different geographic areas in Mexico: four strains were collected at Hospital Regional General Ignacio Zaragoza (HRGIZ), Instituto de Seguridad y Servicios Sociales de los Trabajadores del Estado (ISSSTE) in Mexico City, Mexico; one strain at Pediatric ward of the Hospital General de México Eduardo Liceaga (HGM-P) in Mexico City, Mexico and one strain at Hospital Civil de Guadalajara, Fray Antonio Alcalde (HCG) in Guadalajara Jalisco, Mexico (Alcántar-Curiel et al., 2019b). Strains were grown in LB medium overnight at 37°C and stored in glycerol 20% at -80°C until analysis.

### Antimicrobial susceptibility testing

Antimicrobial susceptibility testing was done by the automated VITEK®-2 System. Microdilution broth method was used to determinate MICs of colistin according to Clinical and Laboratory Standards Institute guidelines (CLSI) (Lewis and Pharm, 2023) using *Escherichia coli* ATCC 25922 as quality control strain. We employed the antimicrobial categories as proposed by Magiorakos including the multidrug-resistant (MDR), extensively drug-resistant (XDR), and pandrug-resistant (PDR) phenotypes (Magiorakos et al., 2012).

### Pulsed-field gel electrophoresis

Genotyping of *A. baumannii* strains was determined by Pulsed-Field Gel Electrophoresis (PFGE) as described previously (Naas et al., 2005; Alcántar-Curiel et al., 2014). Briefly, genomic DNA from all strains was digested with ApaI (New England Biolabs, Beverly, MA) embedded in 1% agarose plugs and then subjected to PFGE with a Gene Path system (BioRad) and using lambda ladder PFGE marker (New Englands Biolabs, Beverly) as molecular marker. The PFGE patterns were analyzed using the GelJ Software (Heras et al., 2015), and individual pulse type (PT) were defined according to the interpretative criteria proposed by Tenover (Tenover et al., 1995). The similarity between profiles was calculated using the Dice coefficient (Dice, 1945), every strain with a correlation greater than 85% was considered as member of the same PT.

### Plasmids characterization

Plasmids content in *A. baumannii* isolates was detected using a gentle lysis procedure for both chromosomal and plasmid DNA, followed by separation through electrophoresis using the Eckhardt technique (Eckhardt, 1978; Alcántar-Curiel et al., 2019a). As a source for the high molecular weight DNA bacterial artificial chromosomes (BAC) of 67, 86, 101, 122, 145, and 195 Kb were used (González et al., 2006). 1Kb Plus DNA Ladder (ThermoFisher Scientific) was used as low molecular weight markers.

### Whole-genome sequence analysis

Total DNA from an isolated colony was extracted using the QIAamp® DNA Mini Kit (Qiagen, Hilden, Germany) according to the manufacturer’s instructions. DNA quality and quantity were evaluated by agarose gel electrophoresis. The genome sequencing of the isolates was carried out at of Instituto Nacional de Medicina Genómica (https://www.inmegen.gob.mx/) in Mexico City, Mexico. Samples were sequenced on the Illumina MiSeq platform at 2 x 250 base pair-end read (Mateo-Estrada et al., 2021). Data were assembled using SPAdes 3.13.1 (Bankevich et al., 2012), and genome annotation was performed using Prokka v.1.14.6 (Seemann, 2014). The Multi Locus Sequence Typing (MLST) was performed according to Oxford MLST scheme, as previously described by Bartual et al. (2005) and Pasteur MLST scheme, as previously described by Diancourt et al. (2010). The ST was designated according to the allelic profiles in the PubMLST database (http://pubmlst.org/abaumannii/). Based on the eBURST and neighbour-joining diagram approach generated by the Phyloviz 2.0 program, MLST clonal complexes (CC) and evolutionary relationships between *A. baumannii* strains were defined (Toledano-Tableros et al., 2021). Antibiotic resistance genes (ARGs) were identified using ResFinder 4.1 (Bortolaia et al., 2020). Virulence-associated genes were identified using the virulence factor database VFDB 2.0 (Darmancier et al., 2022). The mobile genetic elements were characterized using plasmidSPAdes v3.11.1 (Antipov et al., 2016) y MOB-suite v3.0.3 (Robertson and Nash, 2018; Robertson et al., 2020).

### Data access

The whole genome sequences have been deposited at GenBank under the accession numbers JAUPJZ000000000, JAUPJY000000000, JAUPJX000000000, JAUPJW000000000, JAUPJV000000000, JAUPJU000000000 (BioProject PRJNA997334). The accession number of the plasmids is OR436916, OR436917, OR436918, OR436919, OR436920, OR436921, OR436922, OR436923.

## Results

### Clinical data and antibiotic susceptibility pattern

The six *A. baumannii* strains investigated in this study were obtained from clinical samples. The strains Aba/76 and Aba/78 were isolated from cerebrospinal fluid, while Aba/75, Aba/77, Aba/79, and Aba/80 were derived from blood samples. Aba/76, Aba/77, and Aba/78 were isolated from patients who were admitted to the Intensive Care Unit, Aba/75 from a patient in the Internal Medicine department, Aba/79 from a Cardiology patient, and Aba/80 from a Pediatrics patient. Regarding comorbidities, Aba/79 and Aba/80 were isolated from patients with catheter-associated bacteremia, Aba/75 from a patient with septic shock, Aba/76 from a patient with an aneurysm, Aba/77 from a patient who had suffered traumatic brain injury, and Aba/79 from a patient with a pituitary microadenoma. Strains Aba/75, Aba/78 and Aba/79 exhibited a PDR profile demonstrating resistance to aminoglycosides, second- and third-generation cephalosporins, fluoroquinolones, tetracyclines, carbapenems and colistin. The remaining three strains Aba/76, Aba/77 and Aba80 displayed an XDR profile, with one of them showcasing resistance to colistin (**Table 1**).

**Table 1:** Antimicrobial susceptibility profiles of six isolates of *A. baumannii* from Mexican hospitals.

No. Isolate- Hospital	Categories^a^ antimicrobial resistance	MIC μg/mL^b^								
		Amikacin	Gentamicin	Cefotaxime	Cefepime	Levofloxacin	Tetracycline	Imipenem	Meropenem	Colistin
Aba/75- HRGIZ	PDR	>128 (R)	>128 (R)	>128 (R)	64 (R)	32 (R)	>128 (R)	8 (R)	8 (R)	16 (R)
Aba/76- HRGIZ	XDR	>128 (R)	>128 (R)	>128 (R)	32 (R)	8 (R)	>128 (R)	4 (I)	8 (R)	1 (S)
Aba/77- HRGIZ/	XDR	32 (I)	2 (S)	>128 (R)	>128 (R)	16 (R)	16 (R)	64 (R)	16 (R)	32 (R)
Aba/78- HRGIZ	PDR	>128 (R)	>128 (R)	>128 (R)	>128 (R)	16 (R)	>128 (R)	128 (R)	>128 (R)	32 (R)
Aba/79- HCG	PDR	32 (I)	32 (R)	>128 (R)	32 (R)	16 (R)	32 (R)	64 (R)	128 (R)	32 (R)
Aba/80- HGM-P	XDR	>128 (R)	>128 (R)	>128 (R)	128 (R)	32 (R)	>128 (R)	>128 (R)	128 (R)	2 (S)

a Multidrug resistance criterion proposed by Magiorakos et al., 2012; MDR, multidrug-resistant; XDR, extensively drug-resistant; PDR, pandrug-resistant.

b MIC, Minimum Inhibitory Concentration; I, intermediate susceptibility; R, resistant; S, susceptible; HRGIZ, Hospital Regional General Ignacio Zaragoza, Instituto de Seguridad y Servicios Sociales de los Trabajadores del Estado, Mexico City; HGM-P, Pediatric ward of the Hospital General de México Eduardo Liceaga, Mexico City; HCG, Hospital Civil de Guadalajara, Fray Antonio Alcalde, Guadalajara, Jalisco.

### The isolates belong to IC2 and IC5

**Figure 1:**
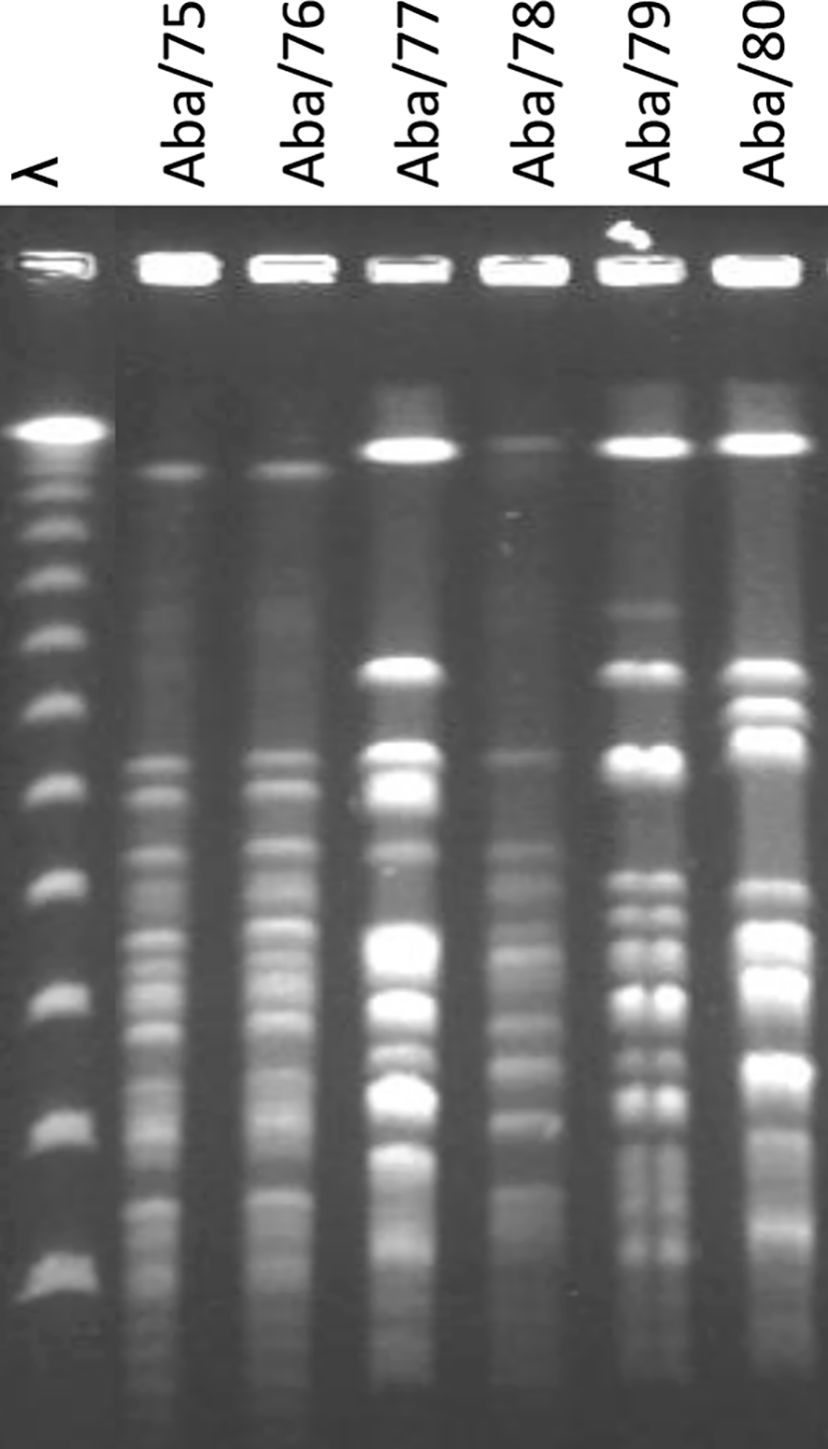
Electrophoretic PFGE pattern of *A. baumannii* strains. λ: Molecular marker.

The isolates were genotyped using PFGE. Among the four strains obtained from ISSSTE, Aba/75 and Aba/76 exhibited macrorestriction patterns with over 85% similarity, both assigned to PT1. We further evaluated the genomic similarity between Aba/75 and Aba/76 using CJ Bioscience’s online Average Nucleotide Identity (ANI) calculator, which revealed a remarkable 99.97% identity in their genomic sequences. Strain Aba/77 was categorized as PT2, while strain Aba/78 was assigned to PT3. Notably, Aba/78 was associated with a nosocomial outbreak. Strain Aba/79 from HCG was designated as PT4, and the strain from HGM, Aba/80, was classified as PT5 (**Figure 1**).

**Figure 2:**
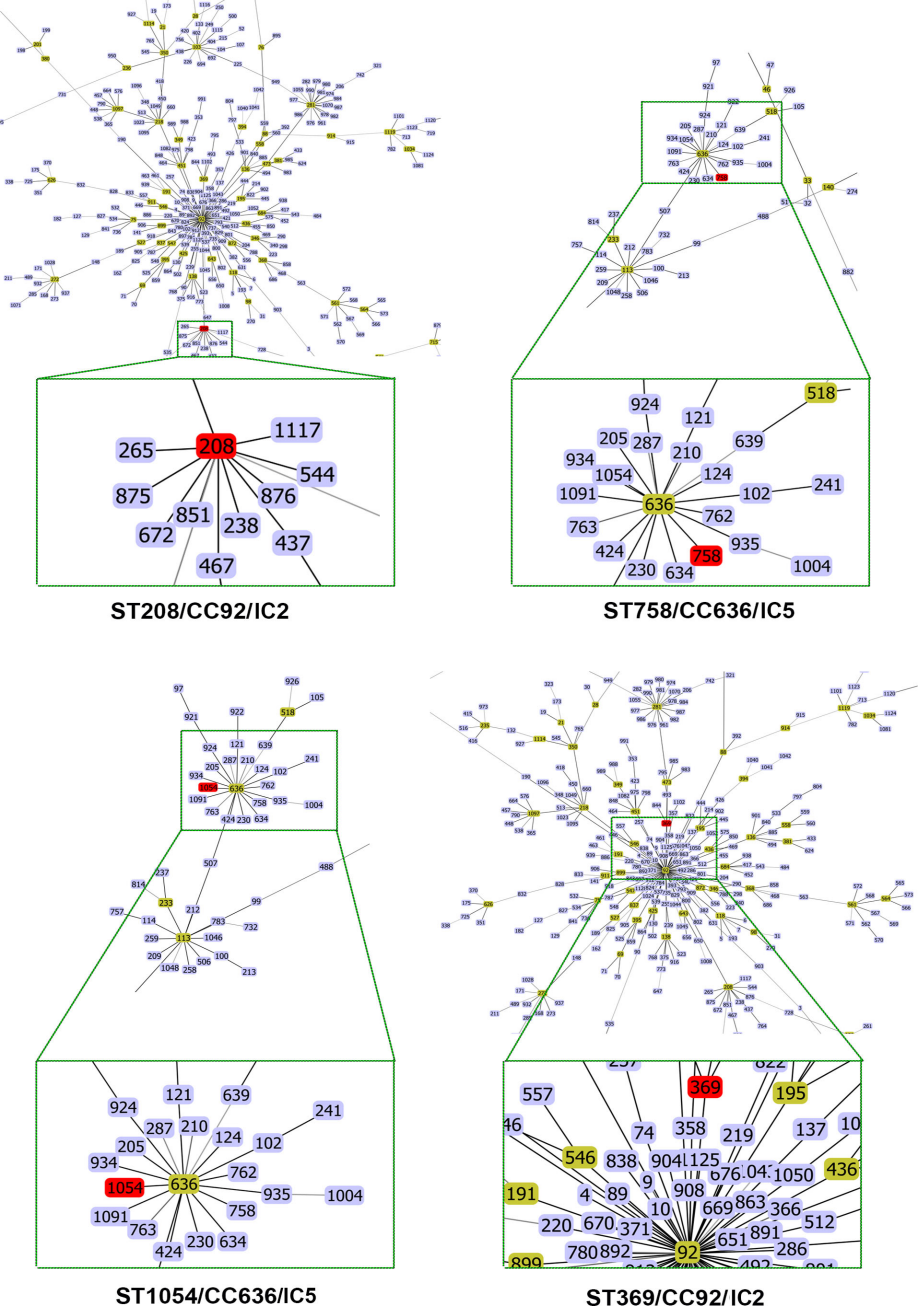
eBurst diagrams for Oxford MLST schemes of *A. baumannii* strains with clonal complexes and STs of close-up.

Pasteur MLST analysis identified that isolates Aba/75, Aba/76, and Aba/78 belonged to ST2, whereas isolates Aba/77, Aba/79, and Aba/80 belonged to ST156. Using the Oxford MLST data, we ascertained that Aba/75 and Aba/76 PT1 strains belonged to ST208, which is part of CC92 within IC2. The strain Aba/77 PT2 and Aba/79 PT4 belonged to ST758, CC636, a member of IC5. The strain Aba/78 PT3 belonged to ST369, CC92, a member of IC2, and strain Aba/80 PT5, belonged to ST1054, CC636, a member of IC5 (**Table 2**). Interestingly, three strains belonged to CC92, a member of IC2, and the other three strains belonged to CC636, a member of IC5 (**Table 2**; **Figure 2**).

**Table 2:** Antibiotic resistance genes in *A. baumannii* strains.

No. strain	Aba/75	Aba/76	Aba/77	Aba/78	Aba/79	Aba/80
Pulse Type/Sequence Type/Clonal Complex/International Clonal	PT1/ST208/CC92/IC2	PT1/ST208/CC92/IC2	PT2/ST758/CC636/IC5	PT3/ST369/CC92/IC2	PT4/ST758/CC636/IC5	PT5/ST1054/CC636/IC5
Total resistance genes	10 genes	10 genes	8 genes	14 genes	7 genes	8 genes
Antibiotic resistance gene	*bla*_ADC-25_	*bla*_ADC-25_	*bla*_ADC-25_	*bla*_ADC-25_	*bla*_ADC-25_	*bla*_ADC-25_
	*bla*_OXA-66_	*bla*_OXA-66_		*bla*_OXA-66_		
			*bla*_OXA-65_		*bla*_OXA-65_	*bla*_OXA-65_
			*bla*_OXA-239_			*bla*_OXA-239_
			*bla*_OXA-72_	*bla*_OXA-72_	*bla*_OXA-72_	
				*bla*_TEM-1_		
	*aph(3’’)-Ib*	*aph(3’’)-Ib*	*aph(3’’)-Ib*	*aph(3’’)-Ib*	*aph(3’’)-Ib*	*aph(3’’)-Ib*
	*aph(6)-Id*	*aph(6)-Id*	*aph(6)-Id*	*aph(6)-Id*	*aph(6)-Id*	*aph(6)-Id*
	*aph(3’)-Ia*	*aph(3’)-Ia*				
						*aph(3’)-VIa*
				*aac(6’)-Ib-cr*		
				*aac(6’)-Ib3*		
				*aadA1*		
	*armA*	*armA*		*arma*		
			*aac(6’)-Ian*		*aac(6’)-Ian*	*aac(6’)-Ian*
	*mph(E)*	*mph(E)*				
	*msr(E)*	*msr(E)*				
	*sul2*	*sul2*	*sul2*		*sul2*	*sul2*
				*sul1*		
				*catB8*		
	*tet(B)*	*tet(B)*		*tet(B)*		
				*qacE*		

*bla*_ADC-25_, Cephalosporinase. *bla*_OXA-66_ and *bla*_OXA-65_, Beta-lactam resistance. *bla*_OXA-239_, and *bla*_OXA-72_, Carbapenem resistance. *bla*_TEM-1_, Beta-lactam resistance. aph(3’’)-Ib, *aph(6)-Id, aph(3’)-Ia, aph(3’)-vIa, aac(6’)-Ib3, aadA1, armA*, and *aac(6’)-Ian, aac(6’)-Ib-cr*, Aminoglycoside resistance. *mph(E)*, Macrolide resistanc. *msr(E)*, Macrolide, Lincosamide and Streptogramin B resistance. *sul1* and *sul2*, Sulphonamide resistance. *catB8*, Phenicol resistance. *tet(B)*, Tetracycline resistance; *qacE*, Disinfectant resistance.

### Resistome and virulome of *A. baumannii* strains

Molecular analysis identified the highest number of ARGs in strain Aba/78 with 14 genes. The strains of the same PT1 Aba/75 and Aba/76 harboured the same number of the ARGs, with 10 genes. Strains Aba/77 and Aba/79 (both ST758) harboured 8 and 7 genes respectively, while strain Aba/80 harboured 8 genes (**Table 2**). The identified ARGs mainly belonged to the families of macrolides, fluoroquinolones, and aminoglycosides. Additionally, genes for resistance to carbapenems (last-line drugs against *A. baumannii*) were identified in all strains.

Analysis of the virulome of six strains revealed the presence of 49 similar genes in all clinical isolates. These genes are involved in adherence, biofilm formation, enzymes, immune evasion, iron uptake, regulation, serum resistance, and stress adaptation (**Supplementary Table 1**). These findings underscore the pathogenic potential exhibited by the majority of strains within the *A. baumannii* complex.

### Plasmids identification, genome rearrangements, and carriage of ARGs

**Figure 3:**
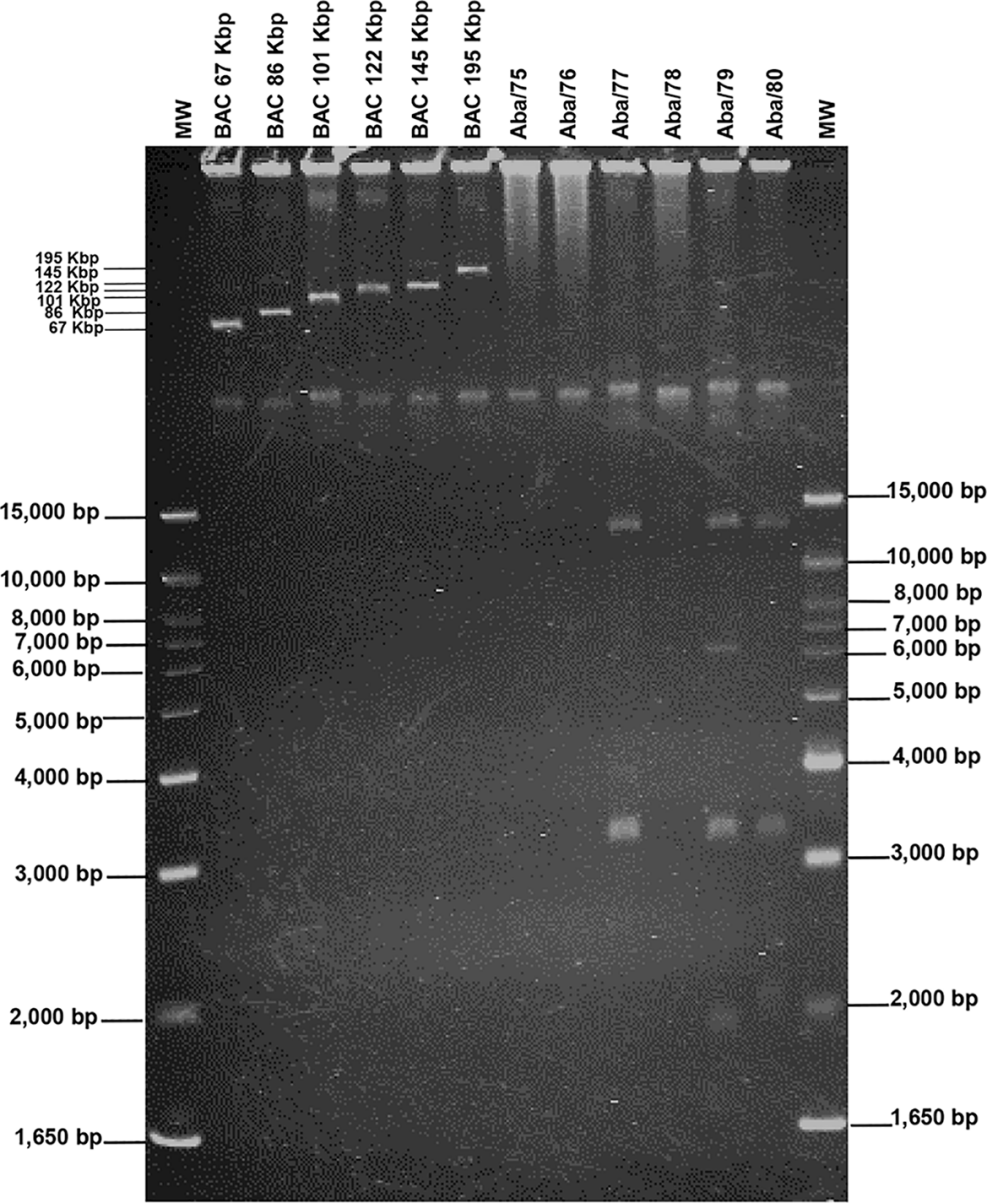
Electrophoretic pattern of plasmids in *A. baumannii* strains. BACs, Bacterial Artificial Chromosome used as reference markers for high molecular weight. MW, Molecular Weight (1KB plus DNA Ladder).

Experimental assays showed that strain Aba/79 had the highest number of replicons, with four plasmids of sizes 14,000 bp, 6,500 bp, 3,200 bp and 1,800 bp. Strain Aba/77 harbour two plasmids with sizes of 14,000 bp and 3,200 bp. Strain Aba/80 harboured three plasmids with sizes of 14,000 bp, 3,200 bp and 2,100 bp. Finally strains Aba/75, Aba/76 and Aba/78 do not harbor plasmids (**Table 3**; **Figure 3**). Based on *in silico* analysis and genome assemblies, four plasmids of sizes 16,610 bp, 10,012 bp, 5,358 bp, and 2,803 bp were identified in the Aba/79 strain. In the Aba/77 strain, two plasmids of sizes 15,362 bp and 5,358 bp were identified, while in the Aba/80 strain, two plasmids of sizes 15,365 bp and 5,358 bp were identified. Additionally, a plasmid of 111,044 bp was detected in the Aba/75 and Aba/76 strains and a plasmid of 10,012 bp in Aba/78 strain (**Table 3**). However, as previously indicated, no plasmids were identified in these three strains through experimental assays.

**Table 3:** Plasmids identified in *A. baumannii* using Gel Eckhardt and the SPAdes Plasmid/Mobrecon programs.

Strain	Aba/75		Aba/76		Aba/77		Aba/78		Aba/79		Aba/80	
Metodology	Eckhardt	PlasmidSPAdes/MOB-suite	Eckhardt	PlasmidSPAdes/MOB-suite	Eckhardt	PlasmidSPAdes/MOB-suite	Eckhardt	PlasmidSPAdes/MOB-suite	Eckhardt	PlasmidSPAdes/MOB-suite	Eckhardt	PlasmidSPAdes/MOB-suite
Plasmids	N/D	111,044	N/D	111,044	14,000	15,362	N/D	10,012	14,000	16,610	14,000	15,365
					3,200	5,358			6,500	10,012	3,200	5,358
									3,200	5,358	2,100	
									1,800	2,803		

The size is expressed in base pairs. N/D, Not detected.

Using ResFinder, the ARGs carried in each replicon were identified. In the case of strain Aba/79, which had four replicons, only the 10,012 bp plasmid harbored the *bla*_OXA-72_ gene, conferring carbapenem resistance, we named this plasmid pAba79f. The plasmid of the same size 10,012 bp in strain Aba/78 also contained the *bla*_OXA-72_ gene (ID GenBank: AY739646), which we named pAba78r. The nucleotide sequence alignment of these two plasmids of the same size that contained the *bla*_OXA-72_ gene showed a 73% close relationship. In these two plasmids, we identified an IS701 sequence located 433 base pairs upstream of the *bla*_OXA-72_ gene. It was found that the plasmids in strains Aba/75, Aba/76 and Aba/80 did not contain any ARGs.

**Figure 4:**
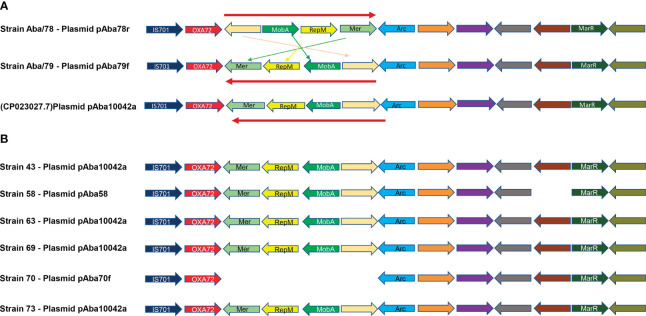
Genetic structure in *A. baumannii* plasmids. **(A)**- Genetic structure of plasmids pAba10042a (CP023027.1), pAba78r and pAba79f. **(B)**- Genetic structure of plasmids identified in *A. baumannii* strains from Mateo-Estrada et al., 2021, pAba10042a, pAba58 and pAba70f.

The analysis of the sequences of both plasmids pAba79f and pAba78r strains using BLASTN (https://blast.ncbi.nlm.nih.gov/Blast.cgi) showed that these plasmids have matched with the circular plasmid pAba10042a of 10,062 bp from *A. baumannii* strain 10042 (ID Gen Bank: NZ_CP023027.1). The analysis of the genome information revealed that both plasmids had 13 coding sequences (CDS) (**Figure 4A**). Among these, six encode hypothetical proteins, one encodes an IS701 transposase, one encodes the carbapenemase *bla*_OXA-72_, one encodes a plasmid mobilization protein MobA/MobB, one encodes a replication inhibitor protein RepM, one encodes a Mer regulatory protein, one encodes a DNA-binding protein Arc, and one encodes a MarR transcriptional regulator.

Although the plasmids pAba79f and pAba78r have the same size and the same number of CDS, their structures are different. A region of 4 CDS which includes a hypothetical protein, MobA, RepM and Mer, is inverted between the two plasmids (**Figure 4A**). The nucleotide sequence of the pAba10042a plasmid obtained from Gen Bank exhibits the same genetic organization as the pAba79f plasmid from Aba/79 strain (**Figure 4A**).

To delve deeper into the dynamics and persistence of plasmids, resistance gene transfer and genetic rearrangements, we selected the genome sequences of five *A baumannii* strains belonging to ST369 (53, 58, 64, 70, 73), from Mateo 2021 et al. and three strains belonging to ST758 (43, 63, 69) sharing the same ST as strains Aba/78 y Aba/79, respectively. Plasmid analysis was conducted in these strains, revealing that strain 73 possesses the pAba10042a plasmid of 10,062 bp, while strain 58 has a plasmid with the same size but a lower number of CDS (13 and 12, respectively), which we named pAba58. Strain 70 harbours a plasmid of 5,464 bp, which we named pAba70F. However, strains 53 and 64 do not have any replicons. As for the three ST758 strains, the pAba10042a of 10,062 bp was identified in all of them. Interestingly, the search for ARGs revealed that all the 10,062 bp and 5,464 bp plasmids harbour the *bla*_OXA-72_ gene (**Figure 4B**). From the analysis of gene structure in the plasmids, we observed that, except for strains 58 and 70, which lack one or more genes, all other strains retained the same structure and orientation as the pAba10042a plasmid (**Figure 4B**). Finally, it is noteworthy that no virulence genes were found in the sequence of the pAba10042a plasmids in each strain.

## Discussion

*A. baumannii* is a bacterium of significant interest and concern in the hospital environment due to its ability to cause HAIs. This pathogen has become a global public health problem due to its increasing antimicrobial resistance, which hinders its treatment and control. One of the main features of *A. baumannii* is its capacity to acquire antimicrobial resistance genes through the lateral transfer of genetic material between different strains, with diverse geographical origins (Graña-Miraglia et al., 2020). In this study, conducted on *A. baumannii* PDR and *A. baumannii* XDR strains from three hospitals and two geographic areas in Mexico, the dissemination of strains with similar PFGE patterns was observed (**Figure 1**). For example, PT1 detected in 2 strains from ISSSTE (Aba/1 and Aba/2), corresponds to clone G previously reported by Alcántar-Curiel in 2019 at ISSSTE, México City, from where these isolates originated. It also corresponds to clone 22 previously reported by Alcántar-Curiel in 2014 strains from HCG, Guadalajara. The distance between these two hospitals is 540 Km. Therefore, we can conclude that this pulse type has been disseminated for at least five years in Mexico.

The eBURST and ST assignment analyses of the strains identified two groups. Group IC2 comprises Aba/75 and Aba/76 of ST208, and Aba/78 of ST369, all of which are members of CC92 (**Figure 2**). Group IC5 includes Aba/77 and Aba/79 of ST758, and Aba/80 of ST1054, all of which are members of CC636 (**Figure 2**). Although ST208 was identified in a strain from ISSSTE in Mexico City, it has been previously identified in San Luis Potosí, México (Tamayo-Legorreta et al., 2016), Guadalajara, Jalisco, México (Mateo-Estrada et al., 2021), and Mexico City (Alcántar-Curiel et al., 2019b; Alcántar-Curiel et al., 2023). Similarly, ST369 identified in a strain from ISSSTE in Mexico City, has been previously described in Nuevo León, Mexico (Bocanegra-Ibarias et al., 2015), Guadalajara, Jalisco, Mexico (Mateo-Estrada et al., 2021), and by our group (Alcántar-Curiel et al., 2019b; Alcántar-Curiel et al., 2023).

The IC5 strains were identified in all three hospitals included in the study. The strains Aba/77 from ISSSTE and Aba/79 from HCG belong to ST758, which has been previously described in four hospitals in Mexico City (Tamayo-Legorreta et al., 2014; Graña-Miraglia et al., 2017; Mancilla-Rojano et al., 2019) and Guadalajara, Jalisco, México (Alcántar-Curiel et al., 2019b; Mateo-Estrada et al., 2021). Recently, in 2020 Graña-Miraglia described that ST758 is an endemic clade that emerged recently within IC5 and exhibits considerable variation in terms of antibiotic resistance genes in Mexican strains. According to the PubMLST database, several studies have reported isolates of this ST in Canada (Loewen et al., 2014), Mexico and Honduras (Graña-Miraglia et al., 2017; López-Leal et al., 2019), and in Colombia (Vanegas et al., 2015). Regarding the Aba/80 strain from HGM, it belongs to ST1054, which has been previously described in other hospitals in Mexico City by Alcántar-Curiel in 2019.

The geographic distribution of IC2 is worldwide, including countries in Latin America (Levy-Blitchtein et al., 2018) and the United States of America (Hujer et al., 2017), which share borders with Mexico. Although this clone has not been described as one of the most relevant in Mexico (Graña-Miraglia et al., 2017; Graña-Miraglia et al., 2020), our results highlight its importance in Mexico. As for IC5, it has mainly spread in Latin America. Studies conducted in Brazil, Argentina, Chile and Paraguay (Nodari et al., 2020) report that the main carbapenemase-producing clones belong to CC15 and CC79, corresponding to IC4 and IC5, respectively (Chagas et al., 2014). Due to its presence in North, Central, and South America, IC5 is considered a Pan-American clone, although it has been described in southeastern Europe (Graña-Miraglia et al., 2020). Our results confirm the dissemination of IC2 and IC5 strains in Mexico, often exhibiting XDR and PDR (Nodari et al., 2020). Therefore, it is suggested to maintain close surveillance in the hospital environment.

A. *baumannii* strains with XDR and PDR are becoming increasingly prevalent in hospital settings. Therefore, it is becoming more crucial to identify the types of ARGs these pathogens harbor to provide targeted and effective therapies that promote patient health.

To address the increasing problem of antimicrobial resistance and safeguard the effectiveness of antimicrobials, studying the resistome is essential. Our data showed significant diversity in the number of ARGs (**Table 2**), highlighting that IC2 strains harbored the highest number of AGRs Aba/78 having 14, and Aba/75 and Aba/76 having 10 each. While IC5 strains contained a lower number of AGRs, Aba/77 had 8, Aba/79 had 7 and Aba/80 had 8. Hernández-González et al. (2022) conducted a genomic study of the resistome using complete genomes of *baumannii* published and reported an average of 29.38 ARGs per genome using the CARD program. This number is higher to the number of AGRs detected in the IC2 strains in this study, a clone that has a longer divergence time and is globally distributed. However, the number of AGRs is lower in the ST758 and ST1054 strains belonging to IC5. The first one has recently expanded as reported by Graña-Miraglia in 2017, while there are few reported cases of the second ST.

Another significant finding was the presence of genes encoding carbapenemases. All strains contained *bla*_OXA-51-like_ genes, which are intrinsic resistance genes, but the allele, differed for each IC; IC2 strains harbored *bla*_OXA-66_, while IC5 strains carried *bla*_OXA-65_. IC5 strains Aba/77 and Aba/80 carried *bla*_OXA-239_ in the chromosome, which belongs to the *bla*_OXA-23-like_ family. Particularly, IC2 strain Aba/78 and IC5 strain Aba/79 shared the presence of the *bla*_OXA-72_ gene, which belongs to the *bla*_OXA-40-like_ family. Notably, the presence of the IS701 element 433 base pairs upstream of *bla*_OXA-72_ strongly supports its association with the carbapenem resistance phenotype. Except for the consistent presence of the *bla*_OXA-72_ gene in both IC groups, these findings imply a selective pattern of cephalosporinases and carbapenemase genes within the strains of IC2 and IC5. This selective trend could serve as a potential determinant in tracing their lineage. Our findings align with Ingti et al.’s study (Ingti et al., 2020), highlighting the coexistence of ADC-type and OXA genes in *A. baumannii*, collectively contributing to resistance against extended-spectrum cephalosporins and carbapenems. Nevertheless, the resistance profile of strain Aba/75 to both carbapenems, and the resistance of strain Aba/76 to meropenem in the absence of carbapenemases, suggests the potential involvement of an alternative resistance mechanism, possibly associated with efflux pump activity or the loss of porins.

The virulome of the *A. baumannii* strains was found to be highly conserved in this study. Forty-nine virulence genes were identified in all strains, and the main categories in which they were grouped included biofilm formation, lipopolysaccharide production, acinetobactin synthesis, and regulatory systems. These mechanisms coincide with those described by (Wong et al., 2017; Harding et al., 2018; Ibrahim et al., 2021), which enable the bacteria to persist in adverse environmental conditions.

The acquisition of genetic material through horizontal transfer in *A. baumannii* is a highly successful process that allows the bacteria to make changes in its genome, even faster than the mutation process (Graña-Miraglia et al., 2017). The diversity in plasmid sizes found in strains of this study is consistent with the sizes described in various studies, ranging from 1.8 kb to over 100 kb, with smaller plasmids being carriers of ARGs generating the most interest (Lean and Yeo, 2017). Gel electrophoresis and *in silico* analysis showed that the number and size of small plasmids are quite similar. However, in the gel electrophoresis assay, the megaplasmids that were identified through *in silico* analysis were not visualized. The limitations of gel electrophoresis lie in the non-identification of large-sized plasmids, probably due to the low copy numbers of the replicon. On the other hand, the limitations of bioinformatic techniques include the updating of program databases, genome coverage, read depth, as well as the presence of repetitive regions that make genome assembly challenging, among others. Significantly, we managed to identify concordances between both methodologies, enabling us to gain insight into the mobile elements contain in *A. baumannii* and determine the resistance genes they harbor.

This is one the first studies where the size of plasmids was compared using both experimental and bioinformatic methodologies, which proved to be complementary. In the Eckhardt method, gel electrophoresis allows each plasmid to be obtained in its linearized form due to the denaturing properties of the system. Meanwhile, the bioinformatic power of *in silico* analysis enabled the identification of plasmids in the genome content. This approach can be of great assistance in future studies concerning plasmid dynamics.

The sequence analysis for ARGs within each of the identified replicons revealed that strains Aba/78 and Aba/79, belonging to ST369/CC92/IC2 and ST758/CC636/IC5, respectively, contained a 10,012 bp plasmid harboring the carbapenem resistance gene *bla*_OXA-72_ in both strains. This plasmid contains 13 coding regions, including the replication and mobilization machinery, allowing it to undergo horizontal transfer to these strains (**Figure 4A**). Despite the significant identity in size and number of identified CDS, these plasmids were not identical to each other. In their structure, they exhibited the inversion of four of their genes involved in plasmid mobilization. The inverted locus is composed of hypothetical protein, followed by MobA, which enables plasmid mobilization, the RepM protein, which serves as an initiator of replication, and the regulatory protein Mer (**Figure 4A**). The sequence of these plasmids showed that the plasmid pAba79f from Aba/79 strain, ST758/CC636/IC5, exhibited 100% identity with plasmid pAba10042a (NZ_CP023027.1) of 10,062 bp (Salgado-Camargo et al., 2020).

To determine whether the genetic structure of these plasmids had been previously identified in reported strains, we selected genomes from a study conducted by Mateo-Estrada in 2021. We included five strains belonging to ST369/CC92/IC2 and three strains from ST758/CC636/IC5, corresponding to Aba/78 and Aba/79 strains respectively. In these genomes, we identified two ST369 strains with a 10,062 bp plasmid and one strain with a 5,464 bp plasmid, and both plasmids contained the *bla*_OXA-72_ gene. As for ST758, we identified three strains carrying a 10,062 bp plasmid with the *bla*_OXA-72_ gene. The sequence of each plasmid showed that four strains harboured the plasmid pAba10042a with identical structures. However, strain 58 had almost the same structure but lacked a hypothetical protein. The plasmid had the same size of 10,062 bp, but in the gene annotation, it only presented 12 of the 13 CDS that strain Aba/79 had. Likewise, strain 70, which carried the 5,464 bp plasmid, lacked the entire locus that was inverted in strains Aba/78 and Aba/79, although the rest of the sequence remained unchanged, including the remaining 9 CDS. This analysis revealed significant plasticity of this plasmid, as it exhibited various structural rearrangements and lengths in several strains. However, in all cases, it retained the *bla*_OXA-72_ resistance gene, which belongs to the serine carbapenemases family *bla*_OXA-24-like_. This gene represents one of the predominant mechanisms for carbapenem resistance in *A. baumannii* (Salgado-Camargo et al., 2020). It is noteworthy that the Aba/78 strain with the inverted locus in the resistance plasmid harboured the highest number of ARGs, totalling 14, including a cephalosporinase and two carbapenemases, and was responsible for a nosocomial outbreak. The epidemiological surveillance of this particular clone is crucial to prevent its spread; importantly, the plasmid it carries can be transferred to strains of different IC.

Our study highlighted the significant ability of *A. baumannii* to persist in the hospital environment for extended periods. Moreover, it revealed the broad range of resistance determinants that *A. baumannii* possesses and the structural versatility of certain plasmids capable of transmitting resistance genes among different clones. Epidemiological studies supported by genetic-molecular data will be increasingly necessary to comprehend the extent of genetic diversity and monitor the transmission of resistance determinants in *A. baumannii* populations.

## Data availability statement

The datasets presented in this study can be found in online repositories. The names of the repository/repositories and accession number(s) can be found in the article/**Supplementary Material**.

## Author contributions

JF-V: Conceptualization, Data curation, Formal analysis, Investigation, Methodology, Software, Writing – original draft. IH-G: Writing – review & editing, Data curation, Software, Methodology. SC-R: Formal analysis, Supervision, Writing – review & editing. MJ-Q: Writing – review & editing, Methodology, Validation. CG-V: Methodology, Formal analysis, Writing – review & editing. VM-E: Methodology, Software, Writing – review & editing. RM-O: Writing – review & editing, Data curation, Methodology. ER-N: Data curation, Methodology, Writing – review & editing. JS-P: Formal analysis, Writing – review & editing. MA-C: Formal analysis, Funding acquisition, Supervision, Writing – original draft, Writing – review & editing.

## Supplementary material

Supplementary Table 1 Virulence factor genes identified in *A. baumannii* strains.
